# Food hygiene knowledge, and practices and their associated factors of street food vendors in Gondar city, Northwest Ethiopia, 2021: A cross-sectional study

**DOI:** 10.1016/j.heliyon.2022.e11707

**Published:** 2022-11-21

**Authors:** Jember Azanaw, Garedew Tadege Engdaw, Hanna Dejene, Samual Bogale, Siraye Degu

**Affiliations:** a Environmental and Occupational Health and Safety, Institute of Public Health, College of Medicine and Health Sciences, University of Gondar, Ethiopia; b Ethiopian Environmental Health professionals, Ethiopia

**Keywords:** Food safety, Food vendors, Knowledge, Attitudes-practice, Ethiopia

## Abstract

**Background:**

Death and illness because of food-borne diseases have greater than previously. According to WHO 2015 report, food-borne diseases affect more than 1/3 of the total population in developing countries each year. Risky food preparation and handling by Street food vendors have made food safety concern for public health. Most individuals nowadays have their meals outside their homes, which are vulnerable to disease caused by contaminated food. This study aimed at assessing the food safety knowledge, and self-reported practices and their associated factors among street food vendors in Gondar city, Northwest Ethiopia.

**Methods:**

A cross-sectional study was conducted to assess food safety knowledge, practices, and their associated factors among 395 street food vendors, which were selected randomly from 700 street food vendors. The data was collected from September 10–28, 2021. Data collection was through face-to-face interview. Then, only fully completed questionnaire were considered for analysis. The data analysis was done using Stata Version 14. Descriptive statistics, binary logistic regression and Spearman's correlation analysis were done. Probability less than to 0.05 was considered statistically significant.

**Results:**

More than half of vendors are licensed (56.5%). Over three-fourths (79.7%) of the food vendors have information about food safety and hygienic practice. Nearly half (50.6%) and 50.9% of study subjects were poor in food hygiene knowledge and practice respectively. Significant relationships were found between knowledge and practice (β1 = 0.46, p < 0.001), and also knowledge and attitude ((β1 = −0.38, p < 0.001). Male food vendors (AOR: 2.05, 95% CI (1.25, 3.10)), food vendors with poor food hygiene attitude (AOR: 2.54, 95% CI (1.65, 3.90)), and those not receive feedback from the customers on food hygiene (AOR: 2.14, 95% CI (1.40, 3.27)) were poor in food hygiene knowledge. Street food vendors who were non-licensed (AOR: 2.06, 95% CI (1.33, 3.17)), no food hygiene information (AOR: 3.03, 95% CI (1.73, 5.31)), and had no training (AOR: 1.26, 95% CI (1.78, 2.04)) were poor in food hygiene practice.

**Conclusion:**

The overall findings of this study indicated that around half of street food vendors’ food hygiene knowledge and practices were poor. Sex, food hygiene attitude, and feedback from customers were significantly associated factors with food hygiene knowledge. In addition, licensing status, food hygiene information, and training related to food hygiene were statistically associated factors with the food hygiene practice. Significant relationships were found between food safety knowledge and food safety practice and also knowledge and attitude.

## 1 Background

Eating foods contaminated with food-derived pathogens and microbial by-product such as toxins can lead to serious illness [1, 2]. In the past few years, death and illness due to foodborne diseases have been greater than before, and food safety has developed into a key concern of healthcare experts and organizations worldwide [3]. Food poisoning is a huge global problem in terms of both human suffering and economic costs [4]. World Health Organization (WHO) estimates that about 600 million people around the world get sick after consuming contaminated food [5]. More than 1/3 of the total population in less developing nations are affected by foodborne diseases each year [6]. The World Bank reports that the economic cost of dangerous food consumption is about $ 110 billion in productivity losses and health care costs in less developed countries [7].

Nowadays, street foods are becoming popular in major towns of Ethiopia including Gondar city. Many people are involved in the preparation and sale of street foods and it becomes common practice around schools, bus stations, and other places where many people are found. But the matter of food safety is not a big deal among street foods vendors [8]. However, food handlers play a principal role in safeguarding food safety and the inhibition of food poisoning since they have direct –indirect contact with foods [9]. Which implies that food poisoning is associated with poor food handling and hygiene practices [10, 11, 12, 13].

Study conducted in Jashore region, Bangladesh, revealed that 72.5% vendors had good knowledge of food safety, only 33% and 0.5% had good food safety attitude and practice, respectively [14]. A study done in Handan city, China indicated that 53.3% stalls did not have direct access to potable water and 73.3% were without adequate hand washing facilities [13]. According to conducted in North Dayi District, Ghana indicated that, 93.6% of street food vendors knew about the washing of hands for 1 min using water and soap before touching food while 68.3% keep ready to eat food at room temperature for 2 h after cooking [15].

The research finding from Ogun State, Nigeria, showed that, only few (18.3%), (18.8%), (15.3%) of the participants cover their hair when they were cooking and serving food, did not handle money while serving, keep long fingernails, respectively [16]. Another study done in Kenya reported that more than half (56.9%) of the street food vendors washed their hands using cold water only and few (20.1%) of them used warm water with soap [17].

The other study conducted in Gojjam Zone, Ethiopia, 51.40% street food vendors had good food safety practice and educational status, monthly income, inspection, training, vending experience were factors showed statistical significance [18]. Previous study in Gondar town half of the vendors (50%) had no frequent hand washing habit with soap and water during the preparation, collecting and displaying of food [19].

Food safety attitudes that are beneficial to food safety have a direct impact on street vendors' food safety practices. A positive attitude towards food safety by food handlers in food safety practices through knowledge during cooking is a potential factor in reducing the risk of food-borne diseases in food facilities [20]. Knowledge is an important predictor of food handlers engaged in participation in hygienic food handling practices [21]. However, food service employees often have diminutive evidence around food contamination hazards and the ways of preventing them [22].

Previous studies in different parts of Ethiopia focused knowledge and practice and their associated factors among food handlers at food establishments [23, 24]. As well as these studies were more focused at bacteriological profile of the foods [19, 25]. Moreover, in some other studies, there were problems in cut of values and sample sizes. Virtually, street food vendors do not fulfill the required requirements for food safety. While street food vendors are expanding time to time as work opportunity. Hence, this study aimed at investigating food hygiene knowledge and practices and their associated factors among street food vendors, which enable in reduction of foodborne disease. The finding of this study will provide better evidence for understanding the level of knowledge, and practice and their predictor variables among street food vendors. After all, this evidence is important to provide insight for policy makers in health, local health professionals in improving food hygiene. This is the original study conducted using the primary data sources collected through face-to-face interview.

### 1.1 Hypotheses and research framework

Hypothesis 1 (H1): Food safety knowledge directly affects self-reported food safety practices. Hence, one of the aims of this study was to evaluate the relationship between food safety knowledge and practice among street food vendors and its direct effect on kitchen hygiene practices, as well as to establish how well are good hygiene practices predicted by knowledge.

Hypothesis 2 (H2): Food safety knowledge is correlated with food safety attitude. Positive attitude in food safety of food handlers practice through knowledge during preparation are factors that may lead to the reduction of the risk of foodborne illnesses [20, 26]. Food vendors' attitude had a positive effect on good hygiene practices like washing hands, and also on cooking practices like washing hands continuously, cover mouth and nose during coughing and sneezing.

**Figure 1:**
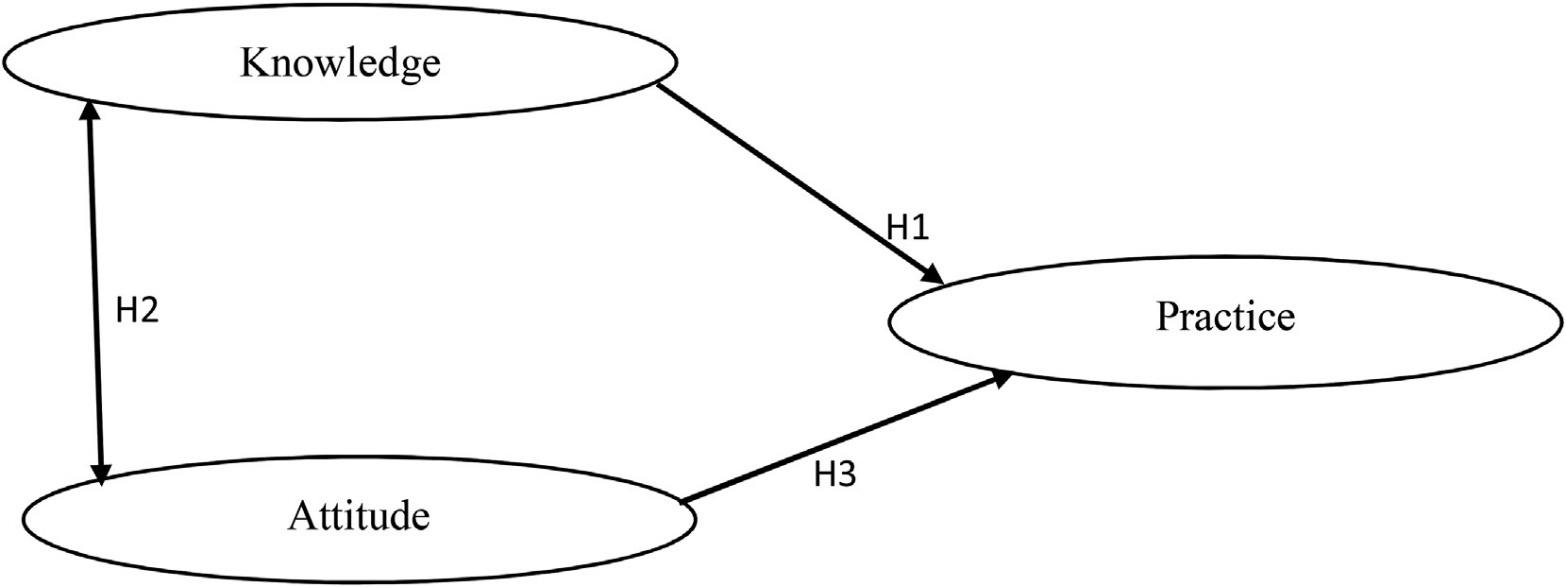
Theoretical Framework.

Hypothesis 3 (H3): Food safety attitude favorable to food safety directly affects street food vendors' self-reported food safety practices. Knowledge was proven to be a significant predictor for engaging adolescents in hygienic food handling procedures [21]. Pearson correlation was used in evaluating the interaction among knowledge, attitude, and practice towards food safety among street food vendors (Figure 1).

## 2 Method

### 2.1 Study design, period, and area

**Figure 2:**
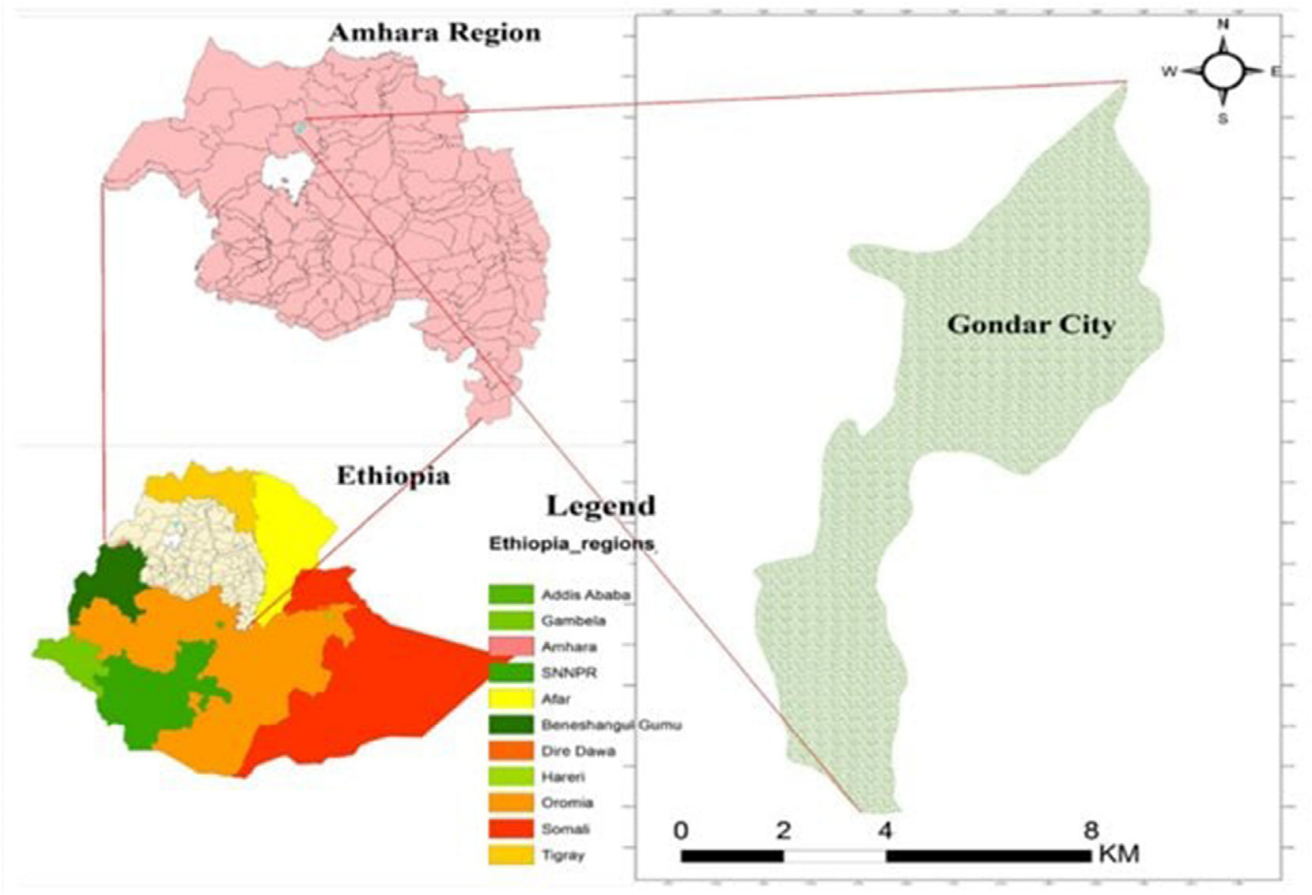
Location of Gondar City.

A cross-sectional study was conducted to assess the knowledge, practices, and associated factors of street vendors in the field of food hygiene from September 10–28, 2021. The study was conducted at Gondar city, Amhara, Northwestern Ethiopia. The city is about 750 km from Addis Ababa, the capital of Ethiopia. The city contains 6 sub-cities (namely Maraki, Azezo Tseda, Arada, Jantekel, Zoble, and Fasil) and 22 Kebeles (the lowest administrative level in Ethiopia). There are many street food vendors in the city, and the number is increasing from time to time. According to 2021 Gondar city Tourism office report, there were around 700 street food vendors. All of the 403 randomly selected street food providers were included in this study (Figure 2).

### 2.2 Sample size and sampling procedure

The sample size of the study street food vendors was determined using a single formula of population proportions based on the following assumptions: The 50% was chosen because no street vendor survey was conducted in the study area. The percentage of knowledge among street vendors is 50%, the confidence level is 95%, Z is 1.96, and 5% non-response rate (Equation 1).

(1) $$n=\frac{{\left(Z_{\frac{\alpha }{2}}\right)}^{2}*P\left(1-P\right)}{d^{2}}\;n=\frac{{\left(1.96\right)}^{2}*\;0.5\left(1-0.5\right)}{{\left(0.05\right)}^{2}}=384$$

[27] Adding a 5% non-responder rate, the final sample size is 403. Street food vendors unevenly distributed in the city. They mostly found around governmental institutions at Arada sub-city (Kebele 5, **7)**, Zoble sub-city (Kebeles 15 and 16) and Maraki sub-city (Kebele 18). Therefore, we have selected study street food vendors using a simple random sampling technique until the convenient sample size is reached.

### 2.3 Questionnaire development and data collection

The questionnaire was created by rewiring related types of literature [28, 29, 30, 31, 32]. The questionnaire used for this survey consisted of four parts.

Part 1: Consists of 12 questions of survey street food vendors ' sociodemographic proles, including age, gender, education level, monthly income, feedback, food hygiene and safety information, and work experience in years.
Part 2: Food safety knowledge was assedon't know for each food safety knowledge question and a score was given for each correct answer. Overall food safety knowledge performance was converted to a percentage by specifying "Yes = 1" and "All other values (no and do not know) = 0" and dividing the total score by the total number of food safety knowledge items [15, 33, 34, 35].
Part 3: Food safety attitudes were assessed by using 12 questions. Attitudes toward food safety were assessed by a ve-level Likert scale question. Regarding the attitude part question, the lowest score (1 point) for "strongly disagree" and the highest score (5 points) for "strongly agree" were given [36, 37].
Part 4: Food safety practices were assessed by using 14 questions. Self-reporting practices were assessed on a five-point Likert scale, from the lowest "never" (1 point) to the highest "always" (5 points) [36, 37].

The total KAP scores below 70% were considered "poor” and scores 70% and higher were considered "good" [38, 39, 40].

Three 4th year environmental health students were involved in this data collection after a day of training. The training was on code of conduct during data collection, the objective of the study and contents of the questionnaire through presentation and pre-test. Five percentage of the questionnaire was pre-tested among non-participants in the final survey to assess the clarity, content, placement, and adequacy of the question. All recommendations were reviewed and used to complete the questionnaire before actual data collection.

As well as reliability of the questionnaire was also assessed using Cronbach's alpha test. The results of Cronbach's alpha were 0.94, and 0.84 for knowledge, and practices questions, respectively. After improvement and reliability check of the tool, content validation is done to checks the questionnaire items it has and their adequateness in measuring variables in the area of food hygiene knowledge and practice.

With the consent of the street food vendors before the start of the survey, respondents can withdraw from participation at any time. Respondents interviewed through face-to-face interview to fill out a questionnaire, and the final completed questionnaire was used for analysis. Statistical Analysis After the collection of the data using the questionnaire, each was checked manually and entered into the EpiInfo Version.7. Then exported to Stata version 14 for analysis. Descriptive statistics such as mean standards, deviations, frequencies, and percentages were used for continuous variables. Level of food safety knowledge (poor/good) and practice (poor/good) were dependent variables of the study. While licensing status (yes/no), sex (male/female), education level, food hygiene information (yes/no), income (500–999, 1000–1500, 1501–5000, ≥5000), food safety attitude (positive/negative), feedback from customers (yes/no), and training (yes/no), were independent variables.

Bivariable binary logistic regression models was used to screen factors associated with food safety knowledge and practices of street food vendors. Variables in bivariable analysis with a p-value less than 0.2 were recruited for multivariable analysis. In that case multivariable analysis, if the p-value is less than 0.05, then association is statistical significant. The strength of association between predictors and outcome variables (level of food safety knowledge and practice) was evaluated by odds ratios with a 95% confidence interval (CI).

Spearman's correlation analysis was also done to show the relationships between knowledge, attitude, and practice in food hygiene. The Pearson correlation test (r) was done to show strength of the associations and corresponding likelihood of errors (p ≤ 5%). The strength was classified as insignificant (0.01–0.09), low (0.10–0.29), moderate (0.30–0.49), considerable (0.5–0.69) and strong (≥0.70) [41].

### 1.4 Ethics approval and informed consent

Ethical clearance was obtained from the Department of Environmental and Occupational Health and Safety, the University of Gondar with Ref. No:EOHS/302/2021. First, respondents were described to and guaranteed that the data collected was treated with utmost privacy, hence it was the duty of the investigator. Then, we provided information on the purpose, procedures, advantages, and disadvantages of the study, and informed written consent was obtained from each study participant. Participation was fully voluntary and partakes were knowledgeable that they could withdraw participation and asking any question not clear at any time of the interview were the rights they have.

## 2 Results

### 2.1 Socio-demographic characteristics of the street food vendors

From 403 samples, 395 street vendors responded to the survey with a 98% response rate. Table 1 shows the demographics of food stall food vendors. More than half of the respondents have a license (56.5%) (Table 1). The majority of street food vendors were female and Orthodox (83.3%) (87.1). Of the street food vendors surveyed, 42.0% (n = 166) were single-marriage history. The majority of street food vendors (67.0%, 265/395) have 1–5 years of street sales experience. Almost one-fifth (6.3%) of street vendors cannot read or write. More than three-quarters (79.7%) of food vendors have information on food safety and hygiene practices, and nearly half (51.6%) of them receive customer feedback (Table 1).

**Table 1:** Socio-demographic characteristics of the food vendors in Gondar city, 2021 (N = 395).

No.	Variables	Category	Frequency	Percentage
Q1.	License status	License	223	56.5
		Not licensed	172	43.5
Q2.	Sex	Female	329	83.3
		Male	66	16.7
Q3.	Age	<20	57	14.4
		21–30	240	60.8
		31–40	81	20.5
		>40	17	4.3
Q4.	Religion (n = 385)	Orthodox	334	84.6
		Catholic	3	0.8
		Protestant	26	6.6
		Muslim	21	5.3
		Other∗	1	0.3
Q5.	Level of education	Not read and write	25	6.3
		Read and write	92	23.3
		Primary school	78	19.7
		Secondary school	139	35.2
		Higher education	61	15.4
Q6.	Marital status	Single	166	42.0
		Married	160	40.5
		Widowed	36	9.1
		Divorced	30	7.6
		Separated	3	0.8
Q7.	Average monthly income	<500	20	5.1
		500–999	162	41.0
		1000–1500	193	48.9
		>1500	20	5.1
Q8.	Vending experience	<1 year	47	11.8
		1–5 years	265	67.0
		>5 years	84	21.2
Q9.	The respiratory problem within 2 weeks	Yes	112	28.4
		No	283	71.6
Q10.	Have food safety and hygiene information	Yes	315	79.7
		No	80	20.3
Q11.	Source of information (n = 315)	Mass media	97	30.8
		Training	50	15.9
		Health extension workers	58	18.4
		Friends	67	21.3
		Parents	43	13.7
Q12.	Get feedback from the costumers	Yes	204	51.6
		No	191	48.4
	1 USD = 47 Ethiopian Birr,∗ = Jewish, Q = Question			

Majority of respondents performed best in the personal hygiene practice (mean score = 97.7 ± 11.4%). Street food vendors asked about how to use the same chopping board for raw and cooked foods related to food contamination issues related to cross contamination. Only 42.5% of respondents knew that using the same chopping board for raw and cooked foods as it has problems with food contamination. Of the total participants, nearly half of the participants responded (50.6%, 46.1%, 55.9% CI) were poor in food hygiene knowledge (Table 2).

**Table 2:** Food hygiene knowledge and frequency with correct answers of street food vendors.

No.	Variables	Correct answers Frequency (%)	Cronbach's Alpha
Q1.	The use of gloves while handling food reduces the risk of food contamination	232 (58.7)	0.94
Q2.	Children, pregnant women, and older individuals are more at risk of food poisoning	320 (81.0)	
Q3.	Do raw foods need to be stored separately from cooked foods?	337 (85.3)	
Q4.	Refrigeration eliminates harmful germs in food	82 (20.8)	
Q5.	Hand washing before cooking reduces the risk of food contamination	361 (91.4)	
Q6.	The diarrheal disease can be transmitted through contaminated food	377 (95.4)	
Q7.	Potentially food contaminant microorganisms are present on human skin	287 (72.7)	
Q8.	Personal hygiene can prevent food contamination	360 (91.1)	
Q9.	Using the same cutting board for raw and cooked foods has no problems with food contamination	168 (42.5)	
Q10.	Foodborne illness can be acquired from the consumption of contaminated food	338 (85.6)	
Q11.	Inadequate cooking of raw food like meat, chicken, and vegetable can cause the outbreak of foodborne illness	307 (77.7)	
Q12.	Cooked foods should be reheated thoroughly	356 (90.1)	
Q13.	Contaminated water can be a vehicle for food contaminants	314 (79.5)	
Q14.	Uncovered abrasion or cuts on fingers and hands can cause food contamination	328 (83.0)	
Total food hygiene knowledge		Poor	200 (50.6%)
		Good	195 (49.4%)

The average score of the questions about towels used to clean food contact surfaces to avoid cleaning hands was 3.64 ± 1.39 for street vendors. The minimum average of street food vendors (1.37 ± 0.71) answered the question of whether washing hands after using the toilet could prevent secondary contamination. Street vendors also showed a positive attitude towards safe foods when it came to washing their hands after using the toilet (95.9%). Knowledge of food safety is important for food handlers (95.7%) (Table 3).

**Table 3:** Percentage, mean scores of questions in food safety attitude towards food safety.

No.	Variables	Percentage	Mean	Std. dev		Cronbach's Alpha	
Q1.	Washing hands after toilet prevent cross-contamination	95.9	1.37	0.71		0.84	
Q2.	Handling food safely is important to food handlers	95.7	1.41	0.73			
Q3.	Consumption of expired food can cause foodborne illness	74.5	1.93	1.17			
Q4.	Defrosted food should not be frozen again	30.1	3.02	1.26			
Q5.	Food and personal hygiene training is important for food handlers	87.4	2.54	1.06			
Q6.	Clean hand towels should be used to wipe hands after washing	72.6	2.07	1.25			
Q7.	The best place to store raw meat or chicken in the refrigerator is on the bottom part	36.2	2.89	1.32			
Q8.	Proper cooking of food could prevent foodborne disease	86.1	1.60	1.08			
Q9.	The food preparation area must be cleaned before and after preparing food	88.4	1.48	0.99			
Q10.	Towel used to clean food contact surfaces should not be used to clean hands	21.3	3.64	1.39			
Q11.	Should not rub your hand on your face, or hair, while working	86.1	1.59	0.98			
Q12.	During coughing or sneezing food handlers need tissue or cloth	90.1	1.56	0.87			
Total food hygiene attitude					Poor		240 (60.8)
					Good		155 (39.2)

The average practical values for washing hands before and after cooking food and not washing eggs before cooking or frying were (4.43 ± 1.00) and (2.46 ± 1.61), respectively. Approximately 80 (81.78%) of the survey street food vendors cleaned and washed the cutting boards, knives, and plates used for raw meat before using them in other foods. Among 395 study street food vendors, nearly half of them (50.9 %, 45.8, 55.7% CI) were poor in food hygiene practice (Table 4).

**Table 4:** Percentage and Mean scores of the correct answer of items in food safety practices.

No.	Variables	Percentage	Mean	Std. dev	Cronbach's Alpha
Q1.	Do you wash your hands before and after cooking food?	88.35	4.43	1.00	0.71
Q2.	Do you keep cooked food at room temperature for a long time?	56.72	2.07	1.51	
Q3.	Do you use your hand to cover your mouth while coughing or sneezing?	73.64	3.98	0.96	
Q4.	Do you wash fruits and vegetables before eating?	75.35	3.94	1.31	
Q5.	Do you read labels with the use by and/or expiry date of packaged food before purchasing?	53.28	3.52	1.25	
Q6.	Do you read the conditions of use and storage of packaged food?	44.56	3.15	1.43	
Q7.	Do you wash eggs before cooking or frying them?	30.88	2.46	1.61	
Q8.	Do you wash and rinse cutting boards, knives, and plates used for raw meat before using them for other food?	81.78	4.15	1.06	
Q9.	Do you wear accessories like rings, and bracelets when cooking food?	38.99	3.09	1.37	
Q10.	Do you use an apron when cooking food?	41.27	3.16	1.46	
Q11.	Do you store raw chicken or meat separately from cooked food?	71.14	3.93	1.30	
Q12.	Do you cover your cut with a bandage and use gloves?	84.30	4.37	0.89	
Q13.	Do you wash your hands before handling raw food?	85.40	4.33	0.93	
Q14.	Do you wash dishes with detergent and water or in a dishwasher after preparing food and before the next use?	87.60	4.42	0.91	
Total food hygiene practice				Poor	201 (50.9)
				Good	194 (49.1)

### 2.2 Interrelationships between food safety knowledge, attitude, and practice

To test the proposed hypothesis of knowledge, attitudes, and practices in food safety were created using Pearson correlation. A P-value ≤ 0.05 was considered statistically significant. Knowledge of food safety has a positive and significant association with food safety practices (β1 = 0.46, p < 0.001). Knowledge of food safety has a negative and significant relationship with attitudes towards food safety (β1 = 0.38, p < 0.001). The results showed that while increasing in knowledge, attitudes towards food safety can decrease by 0.38 units. However, there was no significant association between attitude and practice (p-value = 0.062) (Table 5).

**Table 5:** Pearson correlation of KAP in food safety among street vendors Hypothesis.

Hypothesis	Paths	Standardized estimate	Standard error	95% CI	P value
H1	Knowledge to Food safety practice	0.4555	0.0861	(0.29, 0.63)	0.001
H2	knowledge <–> attitude	-0.3790	0.0725422	(-0.52, -0.24)	0.001
H3	Attitude to Food safety practice	-0.1917	0.0710	(-0.33,-0.05)	0.062

### 2.3 Factors associated with food hygiene knowledge

Sex, food hygiene attitude, and feedback from customers were significantly associated factors with the food hygiene knowledge in the multivariable logistic regression.

The odds of poor food hygiene knowledge were 2.05 times more likely compared to male food vendors (AOR: 2.05, 95% CI (1.25,3.10)). Respondents who have a poor food hygiene attitude were 2.54 (AOR: 2.54, 95% CI (1.65, 3.90)) times more likely to have poor food hygiene knowledge than their counterparts having a good attitude toward food hygiene. Study participants who did not receive feedback from the customers on food hygiene were 2.14 (AOR: 2.14, 95% CI (1.40, 3.27)) times more likely to have poor food hygiene knowledge than the respondents who received feedback on food hygiene (Table 6).

**Table 6:** Bivariable and multivariable results of factors associated with food hygiene knowledge among street food vendors, 2021(N = 395).

Variables		Food hygiene knowledge			COR (95% CI)	AOR (95% CI)
		Poor	Good			
Sex	Male	36		30	1.21 (1.71,3.05)	2.05 (1.25,3.10)∗
	Female	164		165	1	1
Marital status (n = 392)	Single	95		71	1.52 (0.98,2.35)	1.03 (0.78,3.40)
	Windowed	16		20	0.91 (0.81,3.46)	0.86 (0.78,2.52)
	Divorced	14		16	0.99 (0.70,3.34)	1.23 (0.69,2.67)
	Married	75		85	1	1
Food hygiene Attitude	Poor	145		95	2.77 (1.83,4.22)	2.54 (1.65,3.90)∗∗
	Good	55		100	1	1
Feedback from customers about your hygiene practice	No	126		78	2.55 (1.70,3.83)	2.14 (1.40,3.27)∗
	Yes	74		117	1	1
Received any training in food hygiene	No	129		151	0.53 (0.21,2.94)	0.63 (0.39,1.20)
	Yes	71		44	1	1
Food hygiene information	No	35		45	0.71 (0.43,1.16)	0.65 (0.32,2.27)
	Yes	165		150	1	1
Type of food vendor	Ambulatory	122		131	1.14 (0.75,1.71)	1.3 (0.56,2.30)
	Stationary	63		79	1	1
Key:1 = reference, ∗ = p-value at <0.05, ∗∗ p-value at <0.001						

### 2.4 Food hygiene practice predictors

Licensing status, food hygiene information, and training related to food hygiene were statistically associated factors with the food hygiene practice in the multivariable logistic regression.

The odds of poor food hygiene practice were 2.06 times more likely compared to licensed food vendors (AOR: 2.06, 95% CI (1.33, 3.17)). Study participants who did have not food hygiene information were 3.03 (AOR: 3.03, 95% CI (1.73, 5.31)) times more likely to have poor food hygiene practice than their counterparts having food hygiene information. Study participants who did not receive any training in food hygiene were 1.26 (AOR: 1.26, 95% CI (1.78, 2.04)) times more likely to have poor food hygiene practice than their counterparts who received any training in food hygiene (Table 7).

**Table 7:** Bivariable and multivariable results of factors associated with food hygiene practice among street food vendors, 2021(N = 395).

Variables		Food hygiene practice		COR (95% CI)	AOR (95% CI)
		Poor	Good		
Licensing status	Not licensed	104	68	1.99 (1.33,2.98)	2.06 (1.33,3.17)∗∗
	Licensed	97	126	1	1
Sex	Male	36	30	1.20 (2.49,5.43)	1.85 (2.49,5.43)∗
	Female	165	164	1	1
Level of income in ETB	500-999	11	9	2.92 (0.52,6.43)	2.60 (0.79,7.52)
	1000-1500	92	70	2.44 (0.52,3.35)	1.86 (0.75,4.67)
	1501-5000	91	102	1.66 (0.42,2.69)	2.43 (0.65,3.71)
	≥5000	7	13	1	1
Food hygiene information	No	57	23	2.94 (1.73,5.01)	3.03 (1.73,5.31)∗∗
	Yes	144	171		
Received any training in food hygiene	No	155	125	1.86 (1.20,2.89)	1.26 (1.78,2.04)∗
	Yes	46	69	1	1
Type of food vendor	Ambulatory	122	131	1.35 (0.89,2.04)	1.12 (0.75,2.13)
	Stationary	79	63	1	1
Key:1 = reference, ∗ = p-value at <0.05, ∗∗ p-value at <0.001					

## 3 Discussion

Inappropriate the hygienic actions of the food handlers may lead pathogenic microorganisms to spread, stay animated and reproduce to abundant extents to source disease to humans [42]. Street food services acting a significant role in least developing countries like Ethiopia, feeding millions of metropolitan inhabitants daily a wide variety of foods that are relatively cheap and easily accessible [43, 44]. But there are substantial shreds of evidence of health-related difficulties that have been connected with those street foods [45, 46, 47]. Improving and monitoring food safety programs is important to reduce the incidence of food-borne diseases [48]. Therefore the objective of this study is to investigate food hygiene knowledge and practice and their factors associated among street food vendors that are linked with the foodborne disease in Gondar city.

These results indicate that most street food vendors (91.4%) knew the importance of washing their hands before cooking to reduce the risk of food contamination. There is evidence that inadequate personal hygiene, especially inadequate handwashing, is known to be a major risk factor for food contamination leading to food poisoning [49, 50].

Three hundred-eight (83.0%) of street food vendors knew they were found because scratches and cuts on their fingers and hands could contaminate food. This finding was similar to other studies conducted in Qatar, suggesting that 80% of street food vendors covered their hands and fingers while preparing the food [51].

Similar results were found in other studies in which street food vendors provided more accurate answers to questions about good personal hygiene [52]. About cross-contamination (Does raw food need to be stored separately from cooked food? Is it safe to use the same cutting board for raw and cooked food?)

Raw food although isolated from ready-to-eat foods, only 42.5% of respondents have the problem of food contamination on the same cutting board for raw and cooked foods. According to surveys conducted in Johannesburg and Ghana, street food sellers' knowledge of this topic was greater than this result, at 89% and 92%, respectively [53, 54].

Attitude is an important predictor that can affect the performance and practice of food safety for food handlers and, as a result, reduce the incidence of foodborne illnesses [29, 52]. Most of the surveyed street food vendors (95.7%) agreed that knowledge of food safety is important for food handlers. Improper food handling practices were observed in several findings as to the leading cause of foodborne disease in food services establishments [55, 56]. Street food vendors' mean score of washing their hands before and after cooking food was 4.43 ± 1.00 (St.d). To maintain safe food during preparation, personal hygienic practices among the food handlers are important components [51].

According to these results on hand washing practices, 88.35% of the study street food vendors were washed their hands before and after preparing food which was lower than the other prior findings, wherein similarly high scores of 93% [51] and 94.5% [57]. Hand hygiene among food handlers is the most basic critical criterion in safe food handling [58]. Although hand washing is known to be a vital preventive measure in health care scenarios [59], but this works also in the kitchen, for inhibiting the spread of communicable disease through human to human or human to food contact [60, 61].

More than half (56.72%) of study street food vendors keep cooked foods at room temperature for extended periods, which is inconsistent with the very key factors that make foods safer. It is a dangerous habit to leave cooked food in the kitchen for a long time [62].

The majority of street food vendors wash their hands before and after handling food to ensure safe practices such as wearing protective clothing and maintaining work clothes and surface cleanliness. Most of the respondents (87.60%) washed the dishes with detergent and water or in the dishwasher after preparing the food and before using it again, which helped reduce food poisoning. In general, even if the majority KAP related questions are good, some other practices, knowledge, and attitudes questions were lower among study street food vendors that can bring foodborne disease. In general, even if the majority KAP related questions are good, some other practices, knowledge, and attitudes questions were lower among street food vendors that can bring foodborne disease.

### 3.1 The findings on food safety-related KAP correlations

The outcomes of this look at Table 5 revealed that meals protection information does now no longer translate into secure meals dealing with practices. Substantial relationships have been determined between information, exercise, additionally information, and attitude: those have been (p < 0.001) and (p < 0.001) respectively. That there are good-sized relationships between information and exercise is steady with research via way of means of a few preceding researches [63, 64, 65, 66, 67]. The results clearly show that knowledge of food safety has a direct impact on food safety practices.

There was no significant association between attitude and practice (p = 0.062). This finding has been refuted by a similar previous study [29, 39, 68] but it was consistent with other studies [30, 69]. A significant relation was found between knowledge and attitude at p < 0.001: this finding was in line with other previous studies [29, 69] but is inconsistent with other studies done in Malaysia [70]. The finding of this study indicated that food safety knowledge affects directly food safety practice and indirectly through attitude.

### 3.2 Food hygiene knowledge predictors

The current finding revealed that around half (50.6%) of street food vendors had poor knowledge of food hygiene. This result is lower than with studies done in different parts of Ethiopia [71, 72, 73], and India [74]. But this was lower than the finding of the study done among street food vendors regarding food safety knowledge, and practices in the Jashore region, Bangladesh [14].

Sex, food hygiene attitude, and feedback from customers were significantly associated factors with the food hygiene knowledge in the multivariable logistic regression.

In this study, there was a statistically significant difference in food hygiene knowledge between males and females, which indicated that males were poor in food hygiene knowledge than females. This finding was consistent with other previous studies [75, 76]. However, this finding contradicted the study was done in Vietnam [35]. The possible explanation for this variation might be that females get further involved with food handling and may consequently be more knowledgeable [75].

Food hygiene attitude was another important predictor of food safety knowledge among street food vendors. Study subjects with poor attitudes were poor in food hygiene knowledge. This finding was in line with another previous study [71]. This might be due to that attitude involves shaping notions related to the way people think, feel and behave which encompasses a reasoning, responsive, and behavioral component suggesting what should know in the area of food hygiene knowledge [77].

Street food vendors who did not get feedback from customers were more likely in poor food hygiene knowledge. In other words, street food vendors who got the customers' feedback about their drawbacks enhance them in knowledge sharing and make corrective measures for customer gratification.

### 3.3 Food hygiene practice predictors

Of the total study subjects, nearly half of them (50.9 %) were poor in food hygiene practice. This finding was higher than other previous studies conducted in Ghana [15], and Bangladesh [14]. This is comparable with the finding of Dessie town, Ethiopia [78], and Dangila, Ethiopia [73]. These disparities could be due to the difference of the sociodemographic charactestics, study period, study settings.

Licensing status, food hygiene information, and training related to food hygiene were statistically associated factors with the food hygiene practice in the multivariable logistic regression. Study participants without a license were poor in Food hygiene practice as compared with licensed counterparts. This finding was consistent with the study conducted in Addis Ababa, Ethiopia, Ghana, and USA [15, 79, 80]. This might be due to that licensed food vendors' checkups by the regulatory body, training may be given which enhance them to practice good food hygiene and general conditions required from street food vendors. As well since licensed food vendors are subjected to rules and regulations to fulfill criteria by the regulatory body, especially regarding hygiene and sanitation to safeguard customers' health.

Food hygiene-related information looked to be a significant predictor of food hygiene practices. Street Food vendors who have no food hygiene-related information had higher odds of poor food hygiene practice. The possible justification for this could be that food hygiene-related information might aid street food vendors to get improved practice regarding food hygiene as compared to non-informed study participants. The sources of information might be any of the following, media, education, friends, families, and training.

Food hygiene training seemed to be a strong predictor of food hygiene practices. Non-trained street food vendors were poor in food hygiene practices. Similar research results were observed in a study on the effect of training to improve the food hygiene practice amongst food handlers in Ethiopia [71, 76] and street food vendors in Sarawak [81]. This could be that the food safety training may curiously improve the practices of street food vendors. The limitation of this study is the inherent limitation of cross-sectional study design.

## 4 Conclusion

The current findings showed that street food vendors were good in knowledge towards separating cooked foods from raw foods, washing hands before cooking, in uncovered as abrasion or cuts on fingers and hands can cause food contamination. The overall findings of this study indicated that around half of street food vendors’ food hygiene knowledge and practices were poor. Sex, food hygiene attitude, and feedback from customers were significantly associated factors with food hygiene knowledge. In addition, licensing status, food hygiene information, and training related to food hygiene were statistically associated factors with the food hygiene practice. Significant relationships were found between food safety knowledge and food safety practice and also knowledge and attitude. The current result indicated that there is a need improve food safety knowledge and practice among street food vendors. Moreover, this will help in reduce the illness and death related with food-borne diseases.

## Declarations

### Author contribution statement

Jember Azanaw: Conceived and designed the experiments; Performed the experiments; Analyzed and interpreted the data; Contributed reagents, materials, analysis tools or data; Wrote the paper.

Garedew Tadege Engdaw: Conceived and designed the experiments; Performed the experiments; Wrote the paper.

Hanna Dejene; Samual Bogale; Siraye Degu: Performed the experiments; Wrote the paper.

### Funding statement

This work was supported by the 10.13039/501100007861University of Gondar College of Medicine and Health Sciences.

### Data availability statement

The data that has been used is confidential.

### Declaration of interest's statement

The authors declare no conflict of interest.

### Additional information

No additional information is available for this paper.
